# Physical Exercise in Resistant Hypertension: A Systematic Review and Meta-Analysis of Randomized Controlled Trials

**DOI:** 10.3389/fcvm.2022.893811

**Published:** 2022-05-19

**Authors:** Gonzalo Saco-Ledo, Pedro L. Valenzuela, Luis M. Ruilope, Alejandro Lucia

**Affiliations:** ^1^Faculty of Sport Sciences, Universidad Europea de Madrid, Madrid, Spain; ^2^Research Institute of the Hospital Universitario 12 de Octubre (“Imas12”), Madrid, Spain; ^3^Hypertension Unit and Cardiorenal Translational Laboratory, Hospital 12 de Octubre, Madrid, Spain

**Keywords:** office blood pressure, ambulatory blood pressure, nighttime, daytime, hypertensives

## Abstract

Physical exercise reduces blood pressure (BP) in patients with hypertension in general but more evidence is needed specifically for a high-risk phenotype associated with intensive medication, resistant hypertension (RH). In this systematic review and meta-analysis, we aimed to summarize current evidence of the exercise effects on BP in patients with RH. A systematic search was conducted in PubMed, Web of Science and Cochrane Library (from inception to 3rd November, 2021). A random effects meta-analysis was performed when at least two trials assessed the effect of either acute or regular exercise (vs. a control condition) on the same outcome. Ten studies (*N* = 380 participants; 51% female; mean age 52 to 67 years) were included in the review, of which four (*N* = 58) and six (*N* = 322) assessed the effects of acute and regular exercise, respectively. Evidence overall suggests that a single bout of acute exercise results in a short-term (≤ 24 h) reduction of BP, although no meta-analysis could be performed. As for regular exercise, three randomized controlled trials (*N* = 144, 50% female) could be meta-analyzed, which showed that exercise training intervention (8–12 weeks, 3 sessions/week) significantly reduces 24-h (−9.9 mmHg, 95% confidence interval −15.4−4.4 for systolic BP; and −5 mmHg, −7.0−3.0 for diastolic BP) and daytime ambulatory BP (−11.7 mmHg, −17.8−5.7; and −7.4 mmHg, −11.9−2.9). In summary, physical exercise appears as an effective option to reduce BP in patients with RH, although more research is needed to confirm these findings as well as to determine the most effective exercise characteristics.

## Introduction

Approximately 12–15% of hypertensive patients have resistant hypertension (RH) (1), traditionally defined as above-goal clinic (“office”) blood pressure (BP) (i.e., systolic BP (SBP)/diastolic BP (DBP) >130/80 mmHg (2) or >140/90 mmHg (3) according to the American College of Cardiology/American Heart Association or European Society of Cardiology/European Society of Hypertension guidelines, respectively) despite the concurrent use of three or more antihypertensive drugs — commonly including a diuretic, a long-acting calcium channel blocker, and a blocker of the renin-angiotensin system — at maximum or maximally tolerated oral doses (1). RH also includes patients whose BP achieves target values on ≥4 antihypertensive medications (i.e., 'controlled' RH) (1). Because the management of this condition based solely on medications has proven only partially successful (1), non-pharmacological strategies should also be considered.

Lifestyle, particularly physical exercise, can play an important role in BP management in individuals with hypertension (4). Meta-analytical evidence shows that exercise training intervention reduces not only office (5) but also ambulatory BP (ABP) in these individuals (6), with the latter measure being a stronger predictor of cardiovascular diseases (CVD) and mortality (7). Notably, there is recent meta-analytical evidence that a single bout of acute exercise (8) and regular exercise training (6) induce significant short (≤ 24 h) and mid-term (up to ~6 months) reductions in ABP, respectively, among patients with hypertension in general. Furthermore, exercise has minimal side effects compared with drugs (9) and is considered as effective as most antihypertensive agents to reduce office BP (10). However, although the benefits of both acute and regular exercise on BP are well-established in patients with hypertension in general (5, 6, 8), scarcer evidence is available in the context of RH specifically.

Available evidence on the effects of exercise intervention in individuals with RH shows promising results, as confirmed by some non-systematic reviews (11–14). Different trials have reported a beneficial effect of acute (15, 16) or regular exercise (17–20) on office BP or ABP in patients with RH. However, to the best of our knowledge there has been no previous attempts to systematically synthesize the evidence available on the effects of acute or regular exercise on BP measures in patients with RH. Under this context, we aimed to summarize current evidence of the effects of acute or regular exercise on ABP measures in patients with RH.

## Methods

### Data Sources and Search Strategy

The review protocol is registered in PROSPERO (International Prospective Register of Systematic Reviews) (https://www.crd.york.ac.uk/PROSPERO/; Unique identifier: CRD42021287788). Two researchers (GSL, PLV) independently conducted a systematic search — first by title and abstract, and then by full-text — in PubMed, Cochrane Library and Web of Science from inception to 3rd November 2021 using the following search strategy: (“exercise” OR “physical activity” OR “training”) AND (“blood pressure” OR “BP” OR “SBP” OR “DBP”) AND (“resistant hypertension” OR “resistant hypertensive”). This search was supplemented by a manual review of reference lists from relevant publications. We did not search abstracts, posters, and workshop presentations.

### Study Selection

Eligibility criteria were defined in accordance with the Population, Intervention, Comparison, Outcome and Study Design (PICOS) approach (21). We included studies that met each of the following inclusion criteria:

- Population: Adults diagnosed with RH.
- Intervention: Physical exercise, including both a single acute exercise bout and/or regular exercise training (i.e., for several weeks/months). No restrictions were made regarding the frequency, duration, or length of the exercise interventions.
- Comparison: The comparator was a control condition where participants performed no physical exercise.
- Outcomes: Office or ABP.
- Study design: There were no exclusion criteria regarding the study design.

When two studies included part of the same patients' cohort, only data from the study with more participants were included in the meta-analysis.

### Data Extraction

Two reviewers (GSL, PLV) independently identified for each study the number and characteristics of participants, exercise intervention details, endpoints, and results. Data were extracted as mean (standard deviation) when available. When data were provided as intervention effects or using other measures of dispersion (e.g., standard error, 95% confidence interval), the required information was estimated following published guidelines (22). A specific software (WebPlotDigitizer 4.2, San Francisco, CA) was used to extract data provided as a figure in one study (18).

### Quality Assessment

Two authors (GSL, PLV) independently assessed the methodological quality of the different studies using the Tool for the Assessment of Study Quality and Reporting in Exercise (TEXTES) for chronic exercise interventions (23). For studies assessing the short-term effects of a single bout of acute exercise, we used a modified version of the TEXTES scale as proposed elsewhere (24).

### Statistical Analyses

We performed meta-analyses when a minimum of two studies assessed the effects of either acute or regular exercise on a given outcome. A random-effects (DerSimonian and Laird) meta-analysis was performed to assess the mean difference between exercise and control groups using baseline and post-intervention data. Because none of the included studies provided information on the correlation between baseline and post-intervention BP, we used a Pearson's correlation coefficient (r) value of 0.8, in consistence with previous research (25, 26). Publication bias and heterogeneity across studies was assessed with the Begg's test and the I^2^ statistic, respectively. Sensitivity analyses were performed by removing one study at a time. Analyses were conducted using Comprehensive Meta-analysis 2.0 (Biostat; Englewood, NJ) with α = 0.05.

## Results

### Included Studies

Ten studies (*N* = 380 participants, 51% female, age range 52 to 67 years) were included in the review, of which four (15, 16, 27, 28) (*N* = 58, 50% female) assessed the short-term (≤ 24 h) effects of acute physical exercise and six (17, 18, 20, 29–31) (*N* = 322, 51% female) assessed the mid-term (up to 6 months) effects of exercise training intervention (Table 1, Flowchart available as Supplementary Figure 1). Participants of two studies (19, 32) were enrolled in a larger RCT (18). Therefore, we only considered the study with more participants (18).

**Table 1:** Main characteristics of the included studies.

**Study**	**Number of participants analyzed (mean age, % female)**	**Exercise intervention**	**Design**	**Main intervention effects on office and/or ABP**
**Exercise training intervention (regular exercise)**				
Blumenthal et al. (29)	C-LIFE: *N* = 90 (~54 years, 48% female) SEPA: *N* = 50 (~52 years, 48% female)	**C-LIFE** Diet Behavioral weight management **Supervised exercise**: **Modality**: aerobic training (bicycling and/or walking and eventually jogging) **Total duration**: 4 months **Frequency**: 3 sessions/week **Duration per session**: 50–65 min [10 min of warm-up exercises, 30–45 min of bicycling and/or walking (and eventually jogging)], and 10 min of cool-down exercises **Intensity**: 70-85% of heart rate reserve **SEPA (control condition)** Educational session DASH diet materials, weight loss targets (i.e., ~ 1 lb/week) **Exercise goal**s (i.e., ≥150 min/week of aerobic exercise). Patients in SEPA did not participate in the intensive, structured C-LIFE program	RCT (parallel study)	↓ Office SBP ↓ 24-h and daytime ABP (SBP & DBP) ↓ Nighttime ABP (SBP)
Carvalho et al. (31)	Exercise (1): *N* = 5 (~58 years, 80% female) Exercise (2): *N* = 6 (~61 years, 67% female)	**Modality**: strength exercises (1): neutral rowing, squatting, dumbbell supine, knee extension with ankle weights, dumbbell development, dumbbell curl, knee flexion with ankle weights, standing plantar flexion, triceps pulley, and trunk flexion); aerobic exercises (2): stationary cycling elliptical ergometer, and upper-body cycle ergometer) **Total duration**: 12 weeks **Frequency**: 3 sessions/week **Duration per session**: 50-60 min **Intensity**: 50% of HRmax and 11-13 on the Borg's RPE scale	RCT (parallel study)	↓ 24-h and daytime ABP (SBP & DBP) with (2) — but not with (1)
Cruz et al. (18)	Exercise: *N* = 28 (~54 years, 50% female) Control: *N* = 16 (~52 years, 44% female)	**Modality**: resistance (callisthenic exercises against water resistance) + aerobic (walking) exercises **Total duration**: 12 weeks **Frequenc**y: 3 sessions/week **Duration per session:** 60 min **Intensity**: 11-13 on the Borg's RPE scale	RCT (parallel study)	↓ Office BP and 24-h ABP (SBP & DBP).
Dimeo et al. (20)	Exercise: *N* = 22 (~62 years, 54% female) Control: *N* = 25 (~67 years, 61% female)	**Modality:** aerobic exercise (treadmill walking) **Total duration**: 8–12 weeks **Frequency**: 3 sessions/week **Duration per session**: 30-36 min, including intervals of 3-15 min interspersed with 3-min walking intervals **Intensity**: slightly above the aerobic threshold	RCT (parallel study)	↓ 24-h and daytime ABP (SBP & DBP)
Guimaraes et al. (32)	Exercise: *N* = 16 (~55 years, 50% female)	**Modality:** resistance (callisthenic exercises against water resistance) + aerobic (walking) exercises **Total duration**: 2 weeks **Frequency**: 3 sessions/week **Duration per session**: 60 min **Intensity**: HR between the anaerobic threshold and respiratory compensation point, and 11–13 on the Borg's RPE scale	Non-randomized controlled trial	↓ Office SBP ↓ 24-h and daytime ABP (SBP & DBP) ↓ Nighttime ABP (DBP)
Guimaraes et al. (19)	Exercise: *N* = 16 (~55 years, 50% female) Control: *N* = 16 (~52 years, 63% female)	**Modality:** resistance (callisthenic exercises against water resistance) + aerobic (walking) exercises **Total duration**: 12 weeks **Frequency**: 3 sessions/week **Duration per session**: 60 min **Intensity**: 11–13 on the Borg's RPE scale	RCT (Parallel study)	↓ Office SBP and DBP ↓ 24-h, daytime, and nighttime ABP (SBP & DBP)
Kruk et al. (30)	Exercise: *N* = 27 (~55 years, 59% female)	Recommendations concerning diet and healthy lifestyle including physical activity (lifestyle modification) **Total duration**: 6 months	Non-randomized controlled trial	↓ Office SBP and DBP at 3 months ↓ Office DBP at 6 months
Lopes et al. (17)	Exercise: *N* = 26 (~59 years, 46% female) Control: *N* = 27 (~61 years, 44% female)	**Modality:** aerobic exercise (cycling and/or walking) **Total duration**: 12 weeks **Frequency**: 3 sessions/week **Duration per session**: 60 min **Intensity**: 50 to 70% of VO_2max_ (11-14 on the Borg's RPE scale)	RCT (parallel study)	↓ Office SBP ↓ 24-h and daytime ABP (SBP & DBP)
**Acute exercise**				
Pires et al. (16)	Exercise: *N* = 10 (~60 years, 60% female)	**Modality:** strength (air squat, vertical bench press, seated knee raises, seated row, dorsiflexion and plantar flexion, and shoulder abduction); aerobic (walking); combined exercise (aerobic + strength) **Duration:** 6 exercises with 4 sets of 12 submaximal repetitions and a 1-min interval between sets and exercises (strength); 45 min (aerobic); 25 min of aerobic exercise plus 6 exercises with 2 sets of 12 submaximal repetitions (combined) **Intensity**: moderate intensity (3-5 on the adapted Borg scale) (strength); 50–60% of HRmax (aerobic); 50–60% of HRmax and moderate intensity according to the modified Borg scale (combined).	RCT (cross-over study)	↓ 24-h ABP
Ribeiro et al. (27)	Exercise: *N* = 19 (~58.7 years, 47% female)	**Modality:** aerobic exercise walking **Duration:** 10 min **Intensity:** 3 km/h	Non-randomized controlled trial	↑ Central and peripheral SBP ↓ Central and peripheral DBP
Santos et al. (15)	Exercise: *N* = 20 (~53.8 years, 60% female)	**Modality:** aerobic exercise (cycling, light intensity); aerobic exercise (cycling, moderate intensity) **Duration:** 45 min **Intensity:** 50 and 75% of HRmax (or Borg's RPE equivalent for patients receiving beta-blockers)	RCT (cross-over study)	↓ 19-h ABP (DBP, with borderline significance (*p* = 0.053) for SBP) ↓ Daytime and nighttime ABP (SBP & DBP)
Ukena et al. (28)	Exercise: *N* = 9 (~64.9 years, 21% female) patients with RH without renal sympathetic denervation	**Cardiopulmonary exercise testing** (bicycle exercise in a 45° semi-supine position lying on a reclining ergometer)	RCT (parallel study)	No changes

*ABP, ambulatory blood pressure; BP, blood pressure; C-LIFE, Center-Based Lifestyle Intervention; DASH, Dietary Approaches to Stop Hypertension; DBP, diastolic blood pressure; HR, heart rate; HRmax, maximum heart rate; RCT, randomized controlled trial; RPE, rating of perceived exertion; SBP, systolic blood pressure; SEPA, Standardized Education and Physician Advice; VO_2max_, maximal oxygen uptake. ↑, increase; ↓, decrease*.

One study was excluded because the same patients had also participated in previous published research (33). In addition, another study was excluded because analyses were solely focused on the BP effects of exercise training cessation (34).

### Exercise Intervention

Studies assessing the short-term effects of a single acute exercise bout applied sessions of ~10–45 min of strength exercise [moderate intensity, corresponding to 3–5 on the adapted Borg' 0–10 scale of rating of perceived exertion (RPE)] (16), aerobic [50-75% of maximum heart rate (HRmax) (15), 50–60% of HRmax (16), walking at a speed of 3 km/h (27), incremental cycling test (28)], or combined exercise (aerobic exercise at 50–60% of HRmax and strength exercise at moderate intensity, corresponding to 3–5 on the adapted Borg's 0–10 RPE scale) (16).

As for regular exercise, training programs lasted 2 to 24 weeks and included 3 weekly sessions of ~30-60 min duration. Interventions included aerobic [at an intensity of 70-85% of heart rate reserve (29), 50-70% of peak oxygen uptake (17), or slightly above the aerobic threshold (20)] or combined training (i.e., calisthenics and walking against water resistance in a 30–32°C–heated pool at an intensity corresponding to 11–13 on the Borg's 0–20 RPE scale) (18). Three studies reported adherence to the intervention, which averaged 89–100% (17, 18, 29). No major adverse events were noted (e.g., no excessive hypertensive/hypotensive response) in any of the studies (17, 18, 20).

### Quality Assessment

Both the studies assessing the effects of acute (Supplementary Table 1) and regular exercise (Supplementary Table 2) were of overall good quality (median total score = 6.5 and 11, respectively; quality score = 3 and 3.5; reporting score = 6.5 and 7.5).

### Synthesis

Three of the four included studies that assessed the short-term effects of a single bout of acute exercise found a beneficial effect on at least one BP measure. Two RCT found significant benefits on ABP measures after acute exercise (15, 16). One study lacking a control group found benefits of acute exercise on central and peripheral DBP — but not on SBP (27). Finally, one study failed to report a significant reduction in SPB following cardiopulmonary exercise testing in patients with RH who had not undergone renal sympathetic denervation (28).

As for regular exercise, all six studies (17, 18, 20, 29–31) found significant reductions in office BP or ABP measures after exercise training intervention. Five studies showed significant benefits to 24-hour or daytime ABP measures (17, 18, 20, 29, 32), and one found significant benefits to nighttime ABP measures (29). Four studies (17, 18, 29, 30) reported a significant reduction in office BP measures. Three RCT could be meta-analyzed *(N* = 144; 50% female; mean participants' age ranging from 52 to 67 years; weighted average office BP and ABP of 148/83 mmHg and 134/77 mmHg, respectively) (17, 18, 20). Pooled analyses indicated that exercise training tended to decrease office SBP (p=0.059) while significantly reducing office DBP as well as all the different ABP measures (24-h, daytime, and nighttime SBP/DBP, respectively) with no sign of publication bias (all Begg's test *p* > 0.15) (Table 2). The results of 24-h and daytime ABP remained significant in sensitivity analyses. Due to the differences in study designs (i.e., no control group) (30, 31), inclusion of nutritional interventions together with exercise training (29, 30), and the fact that some participants were also enrolled in a larger RCT (19, 32), we could not meta-analyze more studies.

**Table 2:** Pooled analysis of the effect of exercise training intervention on blood pressure (BP) measures in patients with resistant hypertension.

**Outcome**	**MD (95% CI)**	***p*** **-value**	**I^2^ (%)**	**Significance remains** **in sensitivity analyses**
**Office BP**				
SBP	−17.8 (−36.2, 0.6)	0.059	0.0	No
DBP	−6.1 (−11.7, −0.5)	0.032	4.2	No
**Ambulatory BP**				
24-h SBP	−9.9 (−15.4, −4.4)	<0.001	24.0	Yes
24-h DBP	−5.0 (−7.0, −3.0)	<0.001	0.0	Yes
Daytime SBP	−11.7 (−17.8, −5.7)	<0.001	25.9	Yes
Daytime DBP	−7.4 (−11.9, −2.9)	<0.001	10.7	Yes
Nighttime SBP	−9.9 (−19.6, −0.2)	0.045	19.8	No
Nighttime DBP	−4.5 (−8.0, −1.1)	0.010	0.2	No

*Results are shown as absolute mean difference (MD, in mmHg) along with 95% confidence interval (CI)*.

*DBP, diastolic blood pressure; SBP, systolic blood pressure*.

## Discussion and Conclusion

This is the first systematic review and meta-analysis of the exercise effects on BP in patients with RH. Our findings overall suggest that a single bout of acute exercise might reduce BP in the short-term (i.e., within ~24 h) in these patients, although no meta-analysis could be performed. Moreover, “chronic” exercise training interventions (e.g., three sessions/week for up to 6 months) seem to induce significant reductions in office and ABP measures. These results might therefore support the role of exercise as an effective co-adjuvant treatment in patients with RH. This finding is of clinical relevance, particularly when considering that these individuals are at high risk of cardiovascular complications (1). In fact, subjects with elevated resting and/or exercise BP show a worse cardiorespiratory fitness — a strong predictor of CVD and associated mortality — than those with normal BP levels (35), and BP reductions considerably lower than those reported here (e.g., −1.0−2.0 mmHg) have been associated with a reduced risk of cardiovascular complications in people with hypertension in general (36, 37).

In line with our findings, a recent meta-analysis showed that a single bout of acute aerobic exercise induces short-term reductions on ABP measures in patients with hypertension (8). To the best of our knowledge, only four studies to date have analyzed the short-term effects of acute exercise on BP in patients with RH (15, 16, 27, 28), although results seem overall promising. Two randomized cross-over studies found a beneficial effect on ABP after different types of acute exercise in patients with RH (15, 16), and Ribeiro et al. found a significant reduction of central and peripheral DBP — but not SBP — after walking for only 10 min using a non-RCT design in a group of patients with RH (27). It must be noted, nonetheless, that Ukena et al. found no significant effects on SPB after cardiopulmonary exercise testing in patients with RH who had not undergone renal sympathetic denervation (28). Unfortunately, due to the differences in study designs and the paucity of available studies, we could not meta-analyze the effects of acute exercise on BP. Further research is thus needed to confirm whether the benefits of acute exercise previously corroborated in hypertensive patients in general also apply to patients with RH specifically.

The reductions of BP observed in the present study in individuals with RH after exercise training intervention are overall in line with those reported by us in a recent meta-analysis, in which we observed that exercise interventions with a duration of eight to 24 weeks decrease 24-h (average reduction of −5.4 and −3.0 mmHg for SBP and DBP, respectively), daytime (−4.5 and −3.2 mmHg), and nighttime ABP (−4.7 and −3.1 mmHg), respectively, in patients with hypertension in general (*N* = 910) (6). However, greater reductions of ABP seem to occur in patients with RH. These differences might be due to the so-called Wilder's principle (38) — that is, exercise induces larger effects in those patients with higher BP at baseline, such as those with the most severe hypertension phenotypes, notably RH. Other factors can also be involved in these differences, notably the lower number of studies included in the present meta-analysis, which could have partly confounded our results, along with the fact that in one study exercise was performed in a heated pool, which can magnify the hypotensive effects of exercise *per se* (18).

Some limitations must be acknowledged, notably the limited number of studies meta-analyzed, which precluded us from performing sub-analyses attending to variables such as the characteristics of the interventions (in terms of exercise modality or total duration of the exercise training programs) or of the participants (notably, in terms of age, sex or medication status). More research is needed in order to identify the most effective exercise characteristics (modality, intensity, duration) for reducing office BP/ABP in patients with RH, as well as to confirm whether exercise training *per se* might allow reducing the number and/or dosage of drugs needed to manage BP in patients with this condition. The latter question is important because a reduction in medication is associated with lower mortality in individuals with RH (39). Finally, the long-term (i.e., more than 6 months) effects of exercise training intervention also remain to be determined.

In conclusion, our results suggest that exercise training interventions (8–12 weeks, 3 sessions per week, ideally combining aerobic activities at light-moderate intensities such as walking or cycling) as well as muscle strengthening sessions (such as light-moderate intensity weight lifting or calisthenics) decrease both “office” and ABP measures, with even a single bout of acute exercise potentially reducing BP within the following ~24 h. Although further high-quality research (e.g., using a RCT design) is needed to confirm these findings as well as to corroborate the beneficial effects of a single bout of acute exercise on BP, physical exercise appears as an overall effective option to induce meaningful BP reductions in patients with RH.

## Author Contributions

AL, GS-L, and PV: study concept and design, methodology, supervision, interpretation of data, and drafting of the manuscript. PV: statistical analysis. All authors critically revised the manuscript for important intellectual content and approved the final version of the manuscript.

## Funding

Research by LR and GS-L is funded by FEDER/Ministerio de Ciencia e Innovación – Agencia Estatal de Investigación, Spain (PID2020-114862RB-I00/AEI/10.13039/501100011033). PV is supported by a Sara Borrell post-doctoral contract by Instituto de Salud Carlos III (CD21/00138). Research by AL is funded by the Spanish Ministry of Science and Innovation (Instituto de Salud Carlos III, Fondo de Investigaciones Sanitarias and Fondos FEDER, grant number PI18/00139).

## Conflict of Interest

The authors declare that the research was conducted in the absence of any commercial or financial relationships that could be construed as a potential conflict of interest.

## Publisher's Note

All claims expressed in this article are solely those of the authors and do not necessarily represent those of their affiliated organizations, or those of the publisher, the editors and the reviewers. Any product that may be evaluated in this article, or claim that may be made by its manufacturer, is not guaranteed or endorsed by the publisher.
