# Supplementary material for: Physical Exercise in Resistant Hypertension: A Systematic Review and Meta-Analysis of Randomized Controlled Trials
Source: Front Cardiovasc Med. 2022 May 19;9:893811. doi: 10.3389/fcvm.2022.893811 (PMC9161026; doi:10.3389/fcvm.2022.893811)
Supplement: Supplementary file 1 [file Data_Sheet_1.pdf]

Supplementary material

Figure S1. Flowchart of literature search.

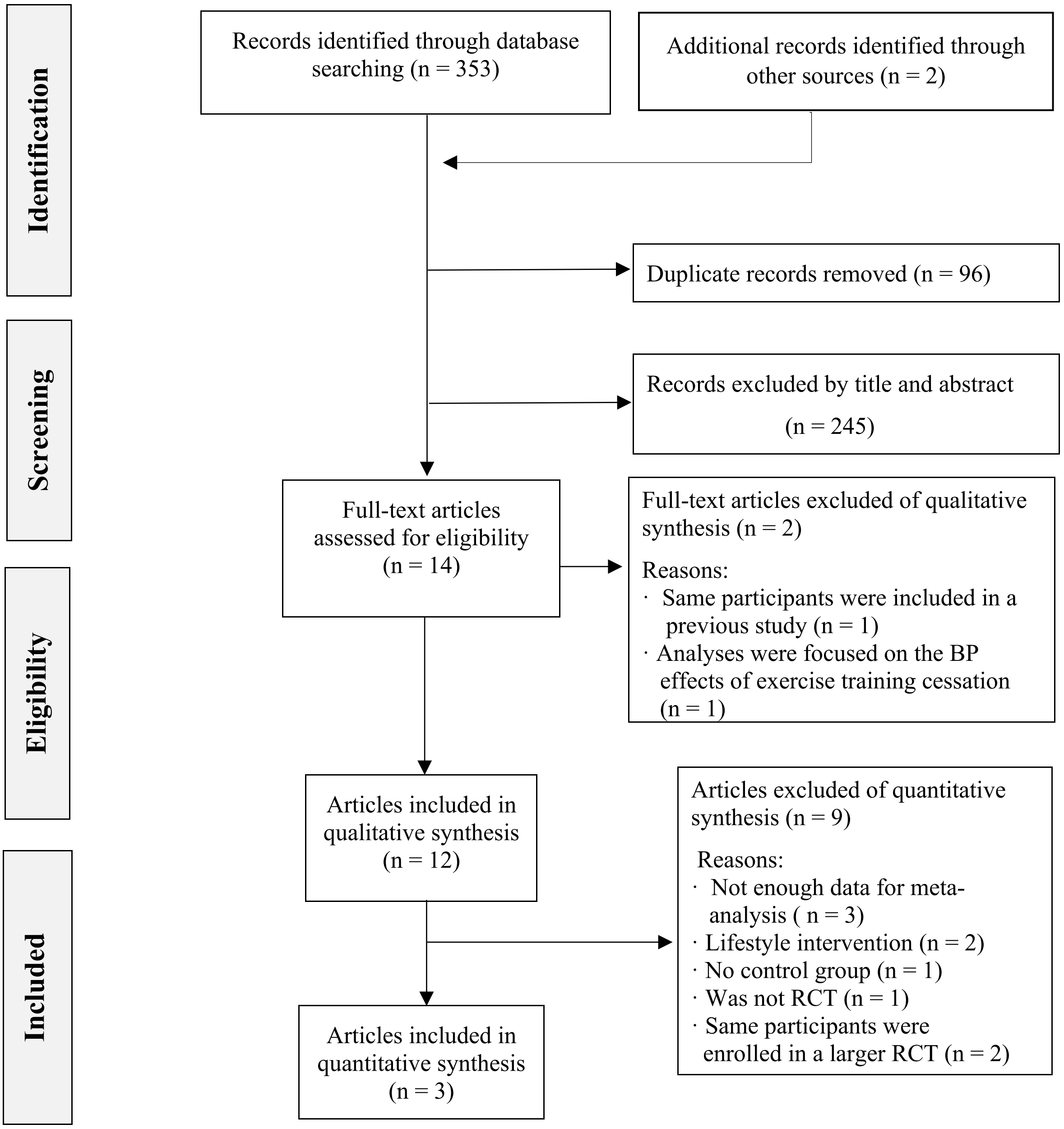

Table S1. Quality of the included studies using the Tool for the Assessment of Study Quality and Reporting in Exercise (TESTEX) scale (version for chronic exercise interventions).

Study	Study quality					Score	Study reporting										Score	Total Score
	1	2	3	4	5	(0-5)	6a	6b	6c	7	8a	8b	9	10	11	12	(0-10)	(0-15)
Blumenthal et al. (29)	1	1	1	1	1	5	1	0	1	1	1	1	1	1	0	1	8	13
Carvalho et al. (31)	1	0	0	0	1	2	1	0	0	1	0	0	1	0	0	1	4	6
Cruz et al. (18)	1	1	0	1	1	4	1	1	1	1	1	1	1	0	0	1	8	12
Dimeo et al. (20)	1	0	0	1	1	3	1	1	1	0	1	1	1	0	0	1	7	10
Guimaraes et al. (32)	1	0	0	1	1	3	1	1	1	1	0	0	1	0	0	1	6	9
Guimaraes et al. (19)	1	1	0	1	1	4	1	1	1	1	1	1	1	0	0	1	8	12
Kruk et al. (30)	1	0	0	0	1	2	1	0	0	1	1	1	1	0	0	0	5	7
Lopes et al. (17)	1	1	1	1	1	5	1	0	1	1	1	1	1	0	1	1	8	13

Study quality: 1 = Eligibility criteria specified; 2 = Randomization specified; 3 = Allocation concealment; 4 = Groups similar at baseline; 5 = Blinding of assessor (for at least one key outcome).

Study reporting: 6 = Outcome measures assessed in 85% of participants (6a = 1 point if completion rate is [85%; 6b = 1 point if adverse events are reported; 6c = 1 point if exercise attendance is reported); 7 = Intention-to-treat analysis; 8 = Between-group statistical comparisons reported (8a = 1 point if between-group statistical comparisons are reported for the primary outcome measure of interest; 8b = 1 point if between-group statistical comparisons are reported for at least one secondary outcome measure); 9 = Point measures and measures of variability for all reported outcome measures; 10 = Activity monitoring in control groups; 11 = Relative exercise intensity remained constant; 12 = all exercise characteristics are reported adequately (i.e. intensity, frequency, mode, duration of session and duration of the intervention) and the exercise volume and energy expenditure can be evaluated.

Table S2. Quality of the included studies using the Tool for the Assessment of Study Quality and Reporting in Exercise (TESTEX) scale (modified version for acute exercise interventions).

Study	Study quality			Score	Study reporting			Score	Total Score
	1	2	3	(0-3)	4	5	6	(0-4)	(0-7)
Pires et al. (16)	1	1	1	3	2	1	1	4	7
Ribeiro et al. (27)	1	0	0	1	0	1	1	2	3
Santos et al. (15)	1	1	1	3	2	1	1	4	7
Ukena et al. (28)	1	1	1	3	2	1	1	4	6

Column numbers correspond to the following criteria: 1: Eligibility criteria specified; 2: Randomization specified; 3: Blinding of assessor (for at least one key outcome); 4: Between-group statistical comparisons reported; 5: Point measures and measures of variability for all reported outcome measures; 6: All exercise characteristics are reported adequately (i.e., intensity, frequency, mode, duration of session and duration of the intervention) and the exercise volume and energy expenditure can be evaluated. Each item could receive a value of '1' or '0' depending on whether it met the criterion or not, except item 4 which could receive a value of '2', '1' or '0' depending on whether between group statistical comparisons were reported for the primary and secondary outcomes of interest, one of the two (primary or secondary), or neither. The score for acute studies ranges from 0 to 7.
